# Identifying quality improvement intervention publications - A comparison of electronic search strategies

**DOI:** 10.1186/1748-5908-6-85

**Published:** 2011-08-01

**Authors:** Susanne Hempel, Lisa V Rubenstein, Roberta M Shanman, Robbie Foy, Su Golder, Marjorie Danz, Paul G Shekelle

**Affiliations:** 1 RAND Corporation, Santa Monica, CA 90407, USA; 2 Veterans Affairs Greater Los Angeles Healthcare System, Los Angeles, CA 90073, USA; 3 David Geffen School of Medicine, Department of Medicine, University of California Los Angeles, Los Angeles, California, USA; 4 School of Public Health, University of California Los Angeles, Los Angeles, California, USA; 5 University of Leeds, Leeds, LS2 9JT, UK; 6 Centre for Reviews and Dissemination, University of York, York, YO10 5DD, UK

## Abstract

**Background:**

The evidence base for quality improvement (QI) interventions is expanding rapidly. The diversity of the initiatives and the inconsistency in labeling these as QI interventions makes it challenging for researchers, policymakers, and QI practitioners to access the literature systematically and to identify relevant publications.

**Methods:**

We evaluated search strategies developed for MEDLINE (Ovid) and PubMed based on free text words, Medical subject headings (MeSH), QI intervention components, continuous quality improvement (CQI) methods, and combinations of the strategies. Three sets of pertinent QI intervention publications were used for validation. Two independent expert reviewers screened publications for relevance. We compared the yield, recall rate, and precision of the search strategies for the identification of QI publications and for a subset of empirical studies on effects of QI interventions.

**Results:**

The search yields ranged from 2,221 to 216,167 publications. Mean recall rates for reference publications ranged from 5% to 53% for strategies with yields of 50,000 publications or fewer. The 'best case' strategy, a simple text word search with high face validity ('quality' AND 'improv*' AND 'intervention*') identified 44%, 24%, and 62% of influential intervention articles selected by Agency for Healthcare Research and Quality (AHRQ) experts, a set of exemplar articles provided by members of the Standards for Quality Improvement Reporting Excellence (SQUIRE) group, and a sample from the Cochrane Effective Practice and Organization of Care Group (EPOC) register of studies, respectively. We applied the search strategy to a PubMed search for articles published in 10 pertinent journals in a three-year period which retrieved 183 publications. Among these, 67% were deemed relevant to QI by at least one of two independent raters. Forty percent were classified as empirical studies reporting on a QI intervention.

**Conclusions:**

The presented search terms and operating characteristics can be used to guide the identification of QI intervention publications. Even with extensive iterative development, we achieved only moderate recall rates of reference publications. Consensus development on QI reporting and initiatives to develop QI-relevant MeSH terms are urgently needed.

## Background

Quality improvement (QI) interventions account for substantial investments by organizations seeking to improve the quality of care. A large volume of literature documents many of these efforts. Advancement in clinical areas often depends heavily on identifying and synthesizing the existing evidence in systematic reviews. To facilitate reviews of QI interventions, the first step is to evaluate electronic search strategies for retrieving relevant articles; inadequate searching reduces the reliability, validity, and utility of all subsequent review steps.

Searches for quality improvement interventions are challenging for a variety of reasons. Researchers have only recently begun to develop a common understanding of quality improvement interventions, to recognize the features that distinguish these from other interventions, and to promote the need for reporting standards [1,2]. Reaching agreement on how to define and apply a common label that sufficiently captures such interventions is difficult [3,4]; quality improvement interventions can cover a diverse range of approaches that variously target patients, healthcare providers, clinical teams, and organizations across clinical fields. While the common goal of the strategies may be to improve how care is delivered in healthcare settings, neither the interventions and intervention components, nor the outcomes are standardized, precluding a simplistic search strategy for identifying interventions [5]. Novel approaches are continually developed and evaluated to meet evolving needs. The outcomes sought to be improved depend on the clinical field and are likely to vary by the target organization. In addition, quality improvement approaches often include multiple intervention components [6].

Databases such as MEDLINE, which is maintained by the National Library of Medicine (NLM), index publications to facilitate the identification of existing evidence. However, no medical subject heading (MeSH) term exists for quality improvement. Thus, whereas the proportion of irrelevant publications identified by typical computerized searches is high, searches for quality improvement publications identify even more such titles. An early study testing individual MeSH terms and text words for the identification of specific quality improvement interventions, such as provider education, showed that the precision of searches varies considerably between individual interventions [7]. A reliable filter is needed to help identify relevant literature while simultaneously screening out irrelevant publications.

Research on search filters has concentrated primarily on methodological and study design related search strategies [8-10]. In subject areas with a broad evidence base, it is common to focus the search by restricting the systematic identification of evidence to a particular study design, most commonly randomized controlled trials (RCTs). Recently, quality improvement search filters ('QI hedges') were published to establish optimal search filters for detecting original studies and reviews on provider and process of care quality improvement interventions, and to detect subsets of 'methodologically sound' studies [11]. Research design restrictions may not be readily applicable to quality improvement publications; a study on a selection of publications deemed crucial for the field of quality improvement included diverse study designs and formats [4].

In the work presented here, we developed, applied, and compared alternative search strategies for finding publications relevant to quality improvement. This investigation of search strategies was part of a larger project aimed at the classification and critical appraisal of quality improvement publications. We aim to facilitate literature syntheses, and expect that future reviews may use parts or all of our approaches to suit specific needs, such as identifying quality improvement interventions for particular conditions, clinical fields, contexts, or outcomes by adding search terms directed at these targets.

## Methods

We developed electronic search strategies for MEDLINE (Ovid interface) and PubMed (access through the NLM and National Institutes of Health (NIH)). MEDLINE is a well-indexed database and usually forms the starting point for search strategies in systematic reviews in healthcare. The Ovid interface provides advanced search functions, such as searching for words in close proximity, while PubMed provides a very user-friendly interface. All searches performed for this analysis were restricted to literature published between inception of the database and January 2008.

In addition, we applied published validated search filters [7,11]. While the QI hedges team-reported full search strategies the earlier work by Balas *et al*. reported on the performance of individual text words and MeSH terms. We combined the intervention and effect variables to test the filter performance.

### Reference sets

To test a search strategy, it is necessary to establish its success in identifying relevant publications. We drew on three sets of publication collections that were deemed pertinent to quality improvement. The relevance of these publications was primarily established outside our working group to ensure that results were not compromised by bias and idiosyncratic definitions of quality improvement. The individual publications included in the sets are shown in the additional file 1.

### Reference set #1: AHRQ

This set comprises a sample of 25 publications classified by two independent raters in a previous project [4] as studies evaluating the effectiveness, impact, or success of a quality improvement intervention. The publications were part of a literature collection deemed by a committee of a 2005 research and evaluation designs and methods conference organized by the Agency for Healthcare Research and Quality (AHRQ) [12] to be highly relevant to the quality improvement field based on each committee member's understanding of quality improvement. The panel members were health services and public health researchers, many of whom had specific programmatic responsibility for developing quality improvement interventions within their organizations, *i.e*., AHRQ, the Centers for Disease Control, the Veterans Administration, the NIH, and the Robert Wood Johnson Foundation.

### Reference set #2: SQUIRE

This set of publications was provided by members of the Standards for Quality Improvement Reporting Excellence (SQUIRE) group. The SQUIRE group was established to provide publishing guidelines for authors of quality improvement interventions. In September 2007, group members nominated papers as a response to a request for exemplar papers in the quality improvement field based on each member's understanding of quality improvement. The selection consisted of 29 publications including intervention evaluations as well as literature reviews. One publication [13] in this set was also included in the AHRQ reference sample (set #1).

### Reference set #3: EPOC

We selected a random sample of 30 publications from all 297 studies registered in November 2007 in a database maintained by the Cochrane Effective Practice and Organization of Care Group (EPOC). EPOC articles are hand searched for this specialized register of evaluations of interventions designed to improve professional practice and the delivery of effective health services, including various forms of continuing education, quality assurance, informatics, financial, organisational, and regulatory interventions that can affect the ability of healthcare professionals to deliver services more effectively and efficiently [14]. Four publications (all conference abstracts) were excluded because they were not indexed in MEDLINE, leaving 26 publications. One publication [15] was also part of the SQUIRE group article selection (set #2).

### Search strategy development and validation

In developing the MEDLINE and PubMed search strategies, we aimed to balance total yield, recall, recall-to-yield ratio, precision, and face validity. We evaluated the total number of records generated by the search strategy (yield). The yield is a feasibility determinant for searches, because resources may limit the search volume that can be screened. The different search strategies and combinations were tested by analyzing the number of reference set publications identified among the search output (recall). We used this measure as an estimate of the sensitivity of the search strategy. We selected a 'best case' strategy based on the recall performance and the recall-to-yield ratio, *i.e*., a strategy that produced both a manageable yield and an acceptable recall rate. A low ratio indicates a disproportionately small recall for the yield. Although the recall performance or sensitivity alone might be promising, the total search volume yielded must be considered to decide whether a strategy is cost-effective.

The search strategy was then applied to obtain a sample of quality improvement publications. The search output was screened by two independent reviewers familiar with the quality improvement literature to determine the number of quality improvement publications within the total output retrieved with the strategy (precision).

The applied search terms were explicitly limited to those that were conceptually relevant to identify a generalizable search strategy (face validity), rather than aiming to find presumably random common denominators within the three reference sample. For example, the index term 'quality of life' was a key word in several SQUIRE group publications (set #2), but the term was not applied because of the lack of generalizability to other quality improvement publications.

### Quality improvement text words

We tested a variety of quality improvement text word-based strategies. For a very simple search strategy, *i.e*., using the terms 'quality' in combination with the word stem 'improv' and 'intervention,' we compared the use of free text words in PubMed with restricting terms to the title, abstract, and MeSH terms (MEDLINE, Ovid). This approach identifies a number of unrelated publications, *e.g*., studies aimed at improving quality of life with any type of intervention. Truncating the terms, *i.e*., using 'improv*' and 'intervention*,' automatically searches variants of the terms. We also investigated the effects of using synonyms for quality improvement interventions, *e.g*., 'quality improvement initiative' or 'quality improvement program.'

### Subject headings

Lacking a quality improvement-specific term, we investigated the use of related and potentially relevant MeSH terms. The selection of MeSH terms was based on screening MeSH terms used in the reference set publications, search strategies from previous projects [16], and by reviewing available MeSH terms on MEDLINE. The selected subject headings were 'quality of health care.sh.,' 'quality assurance, health care.sh.,' 'quality indicators, health care.sh.' and 'health plan implementation.sh.' The use of MeSH terms requires that a publication of interest has been recognized and classified accordingly by database staff, *i.e*., the publication had been assigned a relevant MeSH term in MEDLINE/PubMed. The subject headings were used as indexing terms.

### Intervention components

Although quality improvement initiatives are diverse in nature, they may also be identified by the presence of common quality improvement intervention components [16]. The EPOC group applies a search strategy based on known components of quality improvement[17]. We applied a modification (we did not exclude reviews and meta-analyses) that included: components of promoting change (*e.g*., academic detailing); as well as permanent structural changes (*e.g*., computerized medical records); descriptions of the aim of the initiative (*e.g*., adherence to guidelines); the aim of the initiative (*e.g*., quality assurance) or the aim of the study (*e.g*., program evaluation). Search terms included education, information campaign academic detailing, workshop, training, audit, feedback, dissemination, provider reminders, computerized medical records, fee for service, financial incentives, managed care, discharge planning, guideline implementation, guideline adherence, quality assurance, and program evaluation.

Due to the large number of publications this strategy identified, we combined it with terms to identify evaluations of interventions (including before-after studies, clinical trials, and RCTs).

### CQI methods

Quality improvement approaches are likely to involve continuous quality improvement (CQI) methods; hence we used strategies to develop interventions or to introduce change, such as Plan-Do-Study-Act (PDSA) cycles, to identify quality improvement intervention publications. Terms were generated by interviewing practitioners and evaluators of CQI approaches.

### Search strategy application and precision assessment

We selected a search strategy based on performance across test variables and reference sets and applied it to PubMed. The search was restricted to identify studies published between 2005 and 2007 in ten pertinent journals. The selected journals were *The New England Journal of Medicine, JAMA, Lancet, BMJ, Annals of Internal Medicine, Quality and Safety in Health Care, The American Journal of Managed Care, Medical Care, Health Services Research*, and the *Joint Commission on Quality and Patient Safety*. This subset was based on quality improvement stakeholder recommendations and represents a mixture of the journals that are most relevant and have the highest impact factor.

The search output was screened by two independent raters to identify relevant quality improvement interventions. This inclusion screening was based on each reviewer's implicit understanding of quality improvement rather than a specific agreed definition. This encompassed 'an effort to change/improve the clinical structure, process, and/or outcomes of care by means of an organizational or structural change,' However, as we have shown previously, definitional and subjective interpretation issues are common in this research area [4]. The overall agreement and the kappa statistic were computed for quality improvement publications as well as empirical studies reporting on the effect of interventions, which are usually targeted in evidence syntheses. Studies of effects of interventions were defined as studies reporting empirical data on the success, effectiveness, or impact of a quality improvement intervention [4]. Furthermore, the raters assessed the publications using the Medical Research Council (MRC) framework for complex interventions to identify 'definitive studies' [18]. *Definitive studies*, in contrast to exploratory studies, investigate the effect of an intervention in a suitable research design, typically, but not restricted to, RCTs.

## Results

### Retrieval rates

Table 1 shows the volume of publications produced by each search strategy. The retrieval rate ranged from 2,221 (#9 CQI Text Words) to 216,167 (#7 Intervention components).

**Table 1:** Comparative yields of alternative search strategies

Strategy	Description	Search terms and databases searched	Total retrieval rate (Yield)
#1	QI Text Words, Simple A	1 quality AND improv* AND intervention* (PubMed)	13,572

#2	QI Text Words, Simple B	(quality and improv$ and intervention$).mp (MEDLINE, Ovid)	12,892

#3	QI Text Words, Synonyms A	1 quality 2 improv* OR enhance* 3 intervention* OR initiative* OR strategy* OR program* 4 1 AND 2 AND 3 (PubMed)	35,925

#4	QI Text Words, Synonyms B	1 quality OR system OR process 2 improv* OR enhance* 3 intervention* OR initiative* OR strategy* OR program* 4 1 AND 2 AND 3 (PubMed)	63,593

#5	MeSH terms	1 quality of health care.sh. 2 quality assurance, health care.sh. 3 quality indicators, health care.sh. 4 health plan implementation.sh. 5 1 OR 2 OR 3 OR 4 (MEDLINE, Ovid)	81,733

#6	QI Text Words, Synonyms + MeSH Terms	1 ((quality ADJ3 improv$) or (quality ADJ3 enhanc$)).mp. 2 (quality of health care or quality assurance, health care or quality indicators, health care or health plan implementation).sh. 3 1 AND 2 (MEDLINE, Ovid)	7,750

#7	Intervention Components	1 Intervention components (education, information campaign, academic detailing, workshop, training, audit, feedback, dissemination, provider reminders, computerized medical records, fee for service, financial incentives, managed care, discharge planning, guideline implementation, guideline adherence, quality assurance, or program evaluation) 2 study design filter (randomized controlled trial, controlled clinical trial, intervention study, comparative study, experiment, time series, pre-post test) 3 1 AND 2 (MEDLINE, Ovid)	216,167

#8	QI Text Words, Synonyms + Intervention Components	1 Quality OR (improv* OR enhance*) OR (intervention* OR initiative* OR strategy* OR program*) 2 Intervention component search strategy (education, information campaign, academic detailing, workshop, training, audit, feedback, dissemination, provider reminders, computerized medical records, fee for service, financial incentives, managed care, discharge planning, guideline implementation, guideline adherence, quality assurance, or program evaluation) AND design filter 3 1 AND 2 (MEDLINE, Ovid)	10,895

#9	CQI Text Words	1 pdsa.ti, ab. OR plan-do-study-act.mp. OR plan do study act.mp. OR pdca.ti, ab. OR plan-do-check-act.mp. OR plan do check act.mp. OR define-measure-analyze-improve-control.mp. OR dmaic.ti, ab. OR dmadv.ti, ab. OR define-measure-analyze-design-verify.mp. 2 ((iterative ADJ cycle) OR (rapid ADJ cycle) OR (small ADJ test ADJ2 change)).mp. 3 deming.ti, ab. OR taguchi.ti, ab. OR kansei.ti, ab. Or (six-sigma or (six ADJ sigma)).mp. OR total quality management.ti, ab. Or ((quality ADJ function adj deployment) OR (house ADJ2 quality) OR (quality ADJ circle) OR kaizen.ti, ab. OR (toyota adj production ADJ system).mp. OR (toyota ADJ a3).mp. 4 (breakthrough ADJ series)).mp. ((institute adj2 healthcare ADJ improvement) OR (iso ADJ "9004") OR (iso ADJ 15594*)).mp. OR (IHI OR (Institute ADJ Healthcare adj Improvement)).mp. 5 ((lean ADJ manufacturing) OR (lean ADJ production) OR (lean ADJ healthcare) OR (lean ADJ health adj care) OR (lean ADJ health ADJ service) OR (lean ADJ healthcare ADJ service) OR (lean ADJ health ADJ care ADJ service)).mp. OR ((inventive ADJ problem ADJ solving) OR (inventive ADJ problem-solving) OR (inventive ADJ problemsolving)).mp. OR ((business ADJ process ADJ reengineering) OR (business ADJ process ADJ re-engineering)).mp. OR (system* adj redesign).mp. 6 1 OR 2 OR 3 OR 4 OR 5 (MEDLINE, Ovid)	2,221

#10	Combined Approach	1 (quality ADJ3 improv$).ab, ti. OR (quality ADJ3 enhance$).ab, ti. 2 (quality of health care OR quality assurance, health care OR quality indicators, health care OR health plan implementation).sh. 3 1 OR 2 4 Intervention component search strategy (education, academic detailing, workshop, training, audit, feedback, dissemination, provider reminders, computerized medical records, fee for service, financial incentives, managed care, discharge planning, guideline implementation, guideline adherence, or program evaluation) AND design filter 5 3 AND 4 (MEDLINE, Ovid)	16,535

Search period: database inception to January 2008; *, $ notate truncations; ab.ti/[tiab] indicates term needs to be present in the title or abstract of the publication; .sh indicates MeSH subject heading (not exploded); AND, OR: Boolean operators; ADJ: adjacent function in MEDLINE (Ovid), ADJ3: adjacent terms separated by 3 words or less; mp: term present in the title, original title, abstract, name of substance word, subject heading word, unique identifier; search strategies # 7 and #8 are shown in abbreviated form, the exact PubMed and MEDLINE (Ovid interface) syntax can be obtained from the authors

A simple text word strategy using the truncated key text words for 'improvement,' 'intervention' plus 'quality' (strategy #1 'quality' AND 'improv*' AND 'intervention*') resulted in 13,572 retrieved publications when used as free text words (PubMed). This search identified studies that used the selected terms anywhere in the database record, including the title of the journal that published the study. Restricting the search terms to the title, abstract, or MeSH terms (#2, (quality and improv$ and intervention$).mp; MEDLINE, Ovid) reduced the output to 12,892 publications. By comparison, using only the exact terms without truncation decreased the retrieval rate to 2,924 publications. Omitting the term 'intervention' resulted in a large increase in retrieved publications (truncated: 104,712; exact terms only: 34,362; truncated and limited to title and abstract: 92,358).

Enriching the text words for 'improvement' ('enhance') and 'intervention' ('initiative,' 'strategy,' 'program') through known synonyms more than doubled the search output (strategy #3; 35,925 retrieved publications). Adding further targets of the improvement intervention to the abstract aim 'quality,' *e.g*., system or process improvement, further increased the search output significantly (#4, 63,593 retrieved publications).

In total, 81,733 publications were indexed in MEDLINE (Ovid, #5) with the selected MeSH terms. Quality improvement text words combined with the selected MeSH terms yielded 7,750 publications (#6).

Using common components of quality improvement interventions to identify quality improvement publications produced the largest total retrieval volume even when applying a methodological study design filter (strategy #7; 216,167 publications). Restricting the search to publications that referred to synonyms of quality improvement interventions reduced the output to 10,895 publications (#8).

In total, 2,221 publications on MEDLINE (Ovid) used CQI methods terms such as PDSA cycles (#9) to characterize their intervention approach.

We tested a number of iterations of combined approaches. Applying a search strategy that identified either publications with 'quality improvement' in the title or abstract or publications categorized with the respective MeSH terms, and then restricting the search volume to publications referencing known intervention components identified 16,535 publications (#10).

For comparison, we applied published validated search filters in MEDLINE using the same search period (inception to January 2008) [7,11]. Combinations of the text words and MeSH terms suggested by Balas *et al*. resulted in yields ranging from 1,660 (combining intervention text words and effect variables) to 88,079 (intervention text words). The 'QI hedges' [11] resulted in a yield between 933,460 and 15,691,611. The results are documented in the additional file 2.

### Recall analysis

We evaluated search strategies that yielded a volume of 50,000 publications or fewer in a single database for recall performance relative to our reference publication sets. Table 2 documents the recall results of the strategies and the recall-to-yield ratio, taking the number of recalled reference publications and the total search yield into account to allow a comparison between strategies.

**Table 2:** Recall and recall-to-yield ratio

Search strategy	Recall AHRQ set (n = 25)	Recall SQUIRE set (n = 29)	Recall EPOC set (n = 26)	Recall Across sets	Recall:Yield Ratio
Strategy #1:QI text words, simple (quality AND improv* AND intervention*) (PubMed)	11 (44%)	7 (24%)	16 (62%)	43%	0.00319

Strategy #2: QI text words, simple ((quality AND improv$ AND intervention$).mp) MEDLINE, Ovid)	10 (40%)	5 (17%)	14 (54%)	37%	0.00287

Strategy #3: QI text words, synonyms (PubMed)	12 (48%)	10 (34%)	20 (77%)	53%	0.00148

Strategy #6: QI text words, synonyms AND MeSH terms; (MEDLINE, Ovid)	7 (28%)	6 (21%)	9 (35%)	28%	0.00361

Strategy #8: QI text words, synonyms AND Intervention components (MEDLINE, Ovid)	11 (44%)	9 (31%)	9 (35%)	37%	0.00337

Strategy #9: CQI methods (MEDLINE, Ovid)	2 (7%)	2 (7%)	0 (0%)	5%	0.00210

Strategy #10: Combined approach (MEDLINE, Ovid)	8 (32%)	9 (31%)	14 (54%)	39%	0.00236

* notates truncation; Recall: Number of identified reference set publications; Recall:Yield Ratio: % recall across reference sets divided by total yield

The recall varied across reference sets, but in most, the search strategies identified a third of the reference publications. Overall, strategies showed the best recall for EPOC publications; however, a strategy based on CQI methods did not identify any publication of this reference set. A text word strategy that considered synonyms for improvement and interventions (#3) retrieved 77% of the EPOC publications. The mean recall across sets ranged from 5% (#9, CQI methods) to 53% (#3). The combination of text words plus intervention components (#8) showed the most consistency in identifying publications across all three reference sets; the most variation in recall rates was found for the text word search using known synonyms (#3).

Based on the ratio of recall performance and total retrieval rates, the three best strategies were #6 (0.00361, QI text words, synonyms AND MeSH terms), #8 (QI text words, synonyms, AND intervention components), and #1 (QI text words, simple). Although strategy #3 (QI text words, synonyms) had the highest recall, this performance comes at a price of a high total yield (35,925).

Of the published filters, only two produced a yield of less than 50,000 publications and were evaluated further. The text word filter combining intervention and effect variables designed to retrieve specific quality improvement interventions [7] found none of the publications in the reference sets, the MeSH word based filter identified three publications, which translates to a 4% recall rate across reference sets; the recall-to-yield ratio was 0.00188.

### Precision assessment

We chose the simple text words search strategy ('quality' AND 'improve*' AND 'intervention*') for further analysis. This strategy had shown a manageable total yield, a moderate recall rate, an acceptable recall-to-yield ratio, and high face validity. Applied to PubMed to identify articles published between 2005 and 2007 in the described journals, the search retrieved 183 publications. As a comparison, an application of the text words enriched by synonyms would show a retrieval rate of 357 records, the complex strategy would yield 346 and the MeSH or quality improvement/enhancement strategy would yield 1,171 retrieved records for the same specifications.

Table 3 shows the precision of the search strategy (the number of relevant publications within the total search yield) and the agreement between two independent raters with expertise in quality improvement. At least one of the expert reviewers judged 122 of the 183 publications to be relevant, resulting in a precision estimate of 67%. Conversely, one-third of the identified publications were judged irrelevant by both reviewers. The number of publications rated as relevant by both independent raters was 99 (54%). Reviewer agreement was 87% (total agreement) with a kappa of 0.74.

**Table 3:** Precision and rater agreement

Search strategy 'quality' AND 'improv*' AND 'intervention*'(PubMed, selected journals) Total yield: 183 publications	Precision (n, % relevant publication) N = 183	Total Inter-Rater-Agreement on Relevance	Kappa (95% Confidence Interval)
Publications rated as relevant for quality improvement by at least 1 rater	122 (67%)	---	---

Publications rated as relevant for quality improvement by both raters	99 (54%)	87%	0.74 (CI: 0.64, 0.84)

Publications rated as reporting on effects of a quality improvement intervention by at least 1 rater	74 (40%)	---	---

Publications rated as reporting on effects of a quality improvement intervention by both raters	50 (27%)	90%	0.77 (CI: 0.67, 0.87)

QI Publications rated MRC *definitive study *by at least 1 rater	35 (19%)	---	---

QI Publications rated MRC *definitive study *by both raters	25 (14%)	92%	0.78 (CI: 0.65, 0.91)

* notates truncation; CI: confidence interval

Next, we assessed the number of identified empirical studies reporting on the success, effectiveness, or impact of interventions within the quality improvement intervention publications. Of the total retrieved publications, 74 studies (40%) were classified by at least one reviewer as empirical studies evaluating the effects of a quality improvement intervention. Fifty publications in total were unanimously rated by both raters (90% agreement, kappa 0.77).

Finally, the number of publications reporting on a definitive study, as described in the MRC framework, was 35 (19%) as judged by at least one reviewer. The respective number of studies agreed upon by both raters to be definitive studies was 25 (14%; 92% total agreement, kappa 0.78).

## Discussion

We have compared a variety of search strategies designed to identify quality improvement intervention publications in electronic databases. Overall, these strategies produced moderate results in simultaneously achieving a manageable total yield, as well as acceptable recall, recall-to-yield ratios, and precision.

Although the total retrieval rate varied widely, only one strategy resulted in a yield of fewer than 7,000 publications. Our investigation was restricted to MEDLINE; when adding further pertinent databases to the search, the retrieval rate is likely to double. However, we searched without restricting clinical field, setting, patient characteristic, outcome, or publication year, which represents an uncommon scenario [19-22].

The recall rates ranged from 5% to 53% of identified publications across the three reference sets suggesting only moderate sensitivity. This rate does not reach the standards of methodological search filters [23]. Dickersin *et al*. summarized the proportion of correctly identified references of gold standard reference sets for 18 topics, and reported weighted mean results of 51% of all publications, 77% within journals indexed in MEDLINE, and 63% for selected MEDLINE journals [24]. Search strategies to capture certain study designs, particularly RCTs, are readily available [9], but their level of usage is limited [8,25]. The reported recall rates are approaching other clinical topic filters, for example a strategy to identify palliative care literature had reported sensitivity rates of 65% after modifying an existing search strategy that achieved a 45% rate [26,27]. A study investigating the recall for RCTs of selected interventions, such as physician reminders, reported recall rates of 58% for MeSH terms and 11% for text words. The 'QI hedges' achieved sensitivities of 100% while maintaining a specificity of 89% for identifying evaluations of 'methodologically sound' evaluations of provider interventions [11]. However, by comparison the strategies produce a yield between 933,460 (search strategy: random:.ti, ab. OR educat:.tw. OR exp patient care management) and 15,691,611 (search strategy: control: trial:.mp. OR journal.mp. OR MEDLINE.tw. OR random: trial:.tw) of MEDLINE publications, considerable more than the search strategies presented here.

A further potential explanation for the limited recall rates may lie in the nature of the reference sets. The publication selections of the two expert selected sets were based on each member's understanding of quality improvement rather than an agreed exact and presumably narrower definition. The filter performance was consistently better for the more homogenous EPOC reference set (with the exception of the CQI methods filter); however, the expert selected sets represent the kind of quality improvement publications a variety of stakeholders is interested in retrieving, which can be diverse in nature. Furthermore, the reference sets included between 25 and 29 publications, with a total of 78 unique publications. A study investigating the optimal sample size for bibliographic retrieval studies determined that at least 99 high-quality publications are needed for a 10% or less width of the 95% confidence intervals when developing or validating search strategies [28].

The selected quality improvement publications covered diverse individual interventions with great variation across approaches, research fields, general topics, settings, participants, and methods of delivery. Scrutinizing the individual publications represented in the reference sets there were no unifying themes shared by all articles that could be used as key words in an electronic search. Some publications were so specific that they had no electronically usable identifiers in common with other publications, although expert screeners identified the publications as relevant to quality improvement. A limitation of our study is that the search terms were not selected through a computerized method, and this subjective component may have contributed to the relatively low recall rates in comparison to computer-based methods [9,11]. The individual terms were combined through the Boolean operators 'OR' and 'AND' as well as proximity operators, rather than individually tested and simply combined cumulatively in the final search strategy (*e.g*., term one OR term two OR term three), adding levels of complexity, and the potential for yield and filter failure was simultaneously considered. In addition, our aim in developing the search strategies was generalizability for use in quality improvement literature reviews, rather than maximizing the retrieval of selected reference publications. We explicitly considered the recall-to-yield ratio. Every filter increases the risk of missing pertinent studies. Comprehensive search strategies may identify a large number of relevant studies, but the extent of retrieval volume may be beyond what is conceivably practical.

We identified a simple text word strategy ('quality' AND 'improv*' AND 'intervention*') as the 'best-case' scenario. Although adding synonyms to the chosen terms would have increased the recall rate and presumably the sensitivity, the expected increase in noise caused us to work only with the truncation function of PubMed and MEDLINE (Ovid). However, this feature is limited; some publications [29] were not identified because the authors used the term 'program' instead of 'intervention,' and could be found only by using the known synonym approach. Similarly, intervention components evolve and approaches can only be identified if the feature is known at the time of searching. Given the vast number of ways of describing an intervention and the continuous development of new approaches, the attempt to solve this problem with 'brainstorming' synonyms appears problematic. The CQI term approach did not prove to be fruitful for identifying quality improvement intervention publications. While particular methods may frequently be used in the development of the interventions, these methods do not generally appear in the title or abstract of the publication.

Most of the search terms and strategies we have presented may be of use to facilitate literature syntheses for specific needs. Identifying quality improvement interventions for particular conditions, clinical fields, contexts, or outcomes will limit search volumes, and the key terms, individual strategies, or combinations of strategies may be adopted for more targeted searches. However, the performance of the presented filters is limited, and further research into optimal strategies is required. Validated search strategies are needed in order to be able to evaluate literature reviews and their likely success in covering the universe of pertinent studies; the need for search validations is albeit not specific to quality improvement interventions literature reviews [8].

It is disturbing that, despite our best efforts, we were only moderately successful in identifying pertinent quality improvement interventions. Users of PubMed and MEDLINE depend heavily on the assigned MeSH terms through the NLM. The introduction of a specific MeSH term would significantly facilitate the access to the growing evidence base on quality improvement. Better labeling of publications to ensure identification is also a responsibility of authors. Indeed, the first item of the SQUIRE guidelines suggests the including the term 'quality improvement' in the title of the publication [30]. Without a concerted effort by authors, journals, and medical databases to label quality improvement publications so that they can be identified in literature searches, access to evidence and knowledge accumulation in the field is likely to remain limited.

## Conclusions

The search terms and operating characteristics we have presented can be used to guide the identification of quality improvement intervention publications. Even with extensive iterative development, we achieved only moderate recall rates for reference publications. Consensus development on quality improvement reporting and initiatives to develop quality improvement relevant MeSH terms are urgently needed.

## Competing interests

The authors declare that they have no competing interests.

## Authors' contributions

SH, LR, PS, MD, and RF designed the study. RS, SH, LR, RF, SG, PS, and MD contributed to the search strategy development. LR, PS, MD, and SH inclusion screened the search output in the search strategy application. SH drafted the manuscript, all authors read and approved the final manuscript.

## Supplementary Material

Additional file 1 **Appendix 1**. Reference sets. Click here for file

Additional file 2 **Appendix table**. Application of published validated search strategies. Click here for file

## References

[B1] Batalden PB Davidoff F What is 'quality improvement' and how can it transform healthcare? Qual Saf Health Care 2007 16 2 3 10.1136/qshc.2006.022046 17301192 PMC2464920

[B2] Davidoff F Batalden P Toward stronger evidence on quality improvement. Draft publication guidelines: the beginning of a consensus project Qual Saf Health Care 2005 14 319 325 10.1136/qshc.2005.014787 16195563 PMC1744070

[B3] Danz MS Rubenstein LV Hempel S Foy R Suttorp M Farmer MM Shekelle PG Identifying quality improvement intervention evaluations: is consensus achievable? Qual Saf Health Care 2010 19 279 283 10.1136/qshc.2009.036475 20630931

[B4] Rubenstein LV Hempel S Farmer M Asch DA Yano EM Dougherty D Shekelle P Finding order in heterogeneity: types of quality improvement publications Qual Saf Health Care 2008 17 403 408 10.1136/qshc.2008.028423 19064654

[B5] Michie S Fixsen D Grimshaw JM Eccles MP Specifying and reporting complex behaviour change interventions: the need for a scientific method Implement Sci 2009 4 40 10.1186/1748-5908-4-40 19607700 PMC2717906

[B6] Glasziou P Chalmers I Altman DG Bastian H Boutron I Brice A Jamtvedt G Farmer A Ghersi D Groves T Taking healthcare interventions from trial to practice BMJ 2010 341 c3852 10.1136/bmj.c3852 20709714

[B7] Balas EA Stockham MG Mitchell JA Sievert ME Ewigman BG Boren SA In search of controlled evidence for health care quality improvement J Med Syst 1997 21 21 32 10.1023/A:1022887224126 9172067

[B8] Jenkins M Evaluation of methodological search filters--a review Health Info Libr J 2004 21 148 163 10.1111/j.1471-1842.2004.00511.x 15318913

[B9] InterTASC Information Specialists' Sub-Group InterTASC Information Specialists' Sub-Group search filter resource http://www.york.ac.uk/inst/crd/intertasc/

[B10] Glanville JM Lefebvre C Miles JN Camosso-Stefinovic J How to identify randomized controlled trials in MEDLINE: ten years on J Med Libr Assoc 2006 94 130 136 16636704 PMC1435857

[B11] Wilczynski NL Haynes RB Optimal search filters for detecting quality improvement studies in Medline Qual Saf Health Care 2010 19 e31 20671080 10.1136/qshc.2010.042432

[B12] Agency for Healthcare Research and Quality Expanding Research and Evaluation Designs to Improve the Science Base for Health Care and Public Health Quality Improvement Symposium. Summary of a meeting held September 13-15, 2005 Agency for Healthcare Research and Quality, Rockville, MD http://www.ahrq.gov/qual/phqisymp/

[B13] Landon BE Wilson IB McInnes K Landrum MB Hirschhorn L Marsden PV Gustafson D Cleary PD Effects of a quality improvement collaborative on the outcome of care of patients with HIV infection: the EQHIV study Ann Intern Med 2004 140 887 896 15172903 10.7326/0003-4819-140-11-200406010-00010

[B14] Cochrane Effective Practice and Organisation of Care Group http://epoc.cochrane.org/scope-our-work

[B15] McClellan WM Millman L Presley R Couzins J Flanders WD Improved diabetes care by primary care physicians: results of a group-randomized evaluation of the Medicare Health Care Quality Improvement Program (HCQIP) J Clin Epidemiol 2003 56 1210 1217 10.1016/S0895-4356(03)00198-7 14680672

[B16] Stone EG Morton SC Hulscher ME Maglione MA Roth EA Grimshaw JM Mittman BS Rubenstein LV Rubenstein LZ Shekelle PG Interventions that increase use of adult immunization and cancer screening services: a meta-analysis Ann Intern Med 2002 136 641 651 11992299 10.7326/0003-4819-136-9-200205070-00006

[B17] (EPOC) CEPaOoCG Cochrane Effective Practice and Organisation of Care Group http://epoc.cochrane.org/

[B18] Anderson R New MRC guidance on evaluating complex interventions BMJ 2008 337 a1937 10.1136/bmj.a1937 18945728

[B19] Alexander JA Hearld LR What can we learn from quality improvement research? A critical review of research methods Med Care Res Rev 2009 66 235 271 10.1177/1077558708330424 19176833

[B20] Schouten LM Hulscher ME van Everdingen JJ Huijsman R Grol RP Evidence for the impact of quality improvement collaboratives: systematic review BMJ 2008 336 1491 1494 10.1136/bmj.39570.749884.BE 18577559 PMC2440907

[B21] Arnold SR Straus SE Interventions to improve antibiotic prescribing practices in ambulatory care Cochrane Database Syst Rev 2005 CD003539 10.1002/14651858.CD003539.pub2 PMC7003679 16235325

[B22] Jamtvedt G Young JM Kristoffersen DT O'Brien MA Oxman AD Audit and feedback: effects on professional practice and health care outcomes Cochrane Database Syst Rev 2006 CD000259 10.1002/14651858.CD000259.pub2 16625533

[B23] Robinson KA Dickersin K Development of a highly sensitive search strategy for the retrieval of reports of controlled trials using PubMed Int J Epidemiol 2002 31 150 153 10.1093/ije/31.1.150 11914311

[B24] Dickersin K Scherer R Lefebvre C Identifying relevant studies for systematic reviews BMJ 1994 309 1286 1291 7718048 10.1136/bmj.309.6964.1286 PMC2541778

[B25] Jenkins M Johnson F Awareness, use and opinions of methodological search filters used for the retrieval of evidence-based medical literature-a questionnaire survey Health Info Libr J 2004 21 33 43 15023207 10.1111/j.1471-1842.2004.00480.x

[B26] Sladek RM Tieman J Currow DC Improving search filter development: a study of palliative care literature BMC Med Inform Decis Mak 2007 7 18 10.1186/1472-6947-7-18 17597549 PMC1931586

[B27] Sladek R Tieman J Fazekas BS Abernethy AP Currow DC Development of a subject search filter to find information relevant to palliative care in the general medical literature J Med Libr Assoc 2006 94 394 401 17082830 PMC1629429

[B28] Yao X Wilczynski NL Walter SD Haynes RB Sample size determination for bibliographic retrieval studies BMC Med Inform Decis Mak 2008 8 43 10.1186/1472-6947-8-43 18823538 PMC2569926

[B29] Wells K Sherbourne C Duan N Unutzer J Miranda J Schoenbaum M Ettner SL Meredith LS Rubenstein L Quality improvement for depression in primary care: do patients with subthreshold depression benefit in the long run? American Journal of Psychiatry 2005 162 1149 1157 10.1176/appi.ajp.162.6.1149 15930064

[B30] Davidoff F Batalden P Stevens D Ogrinc G Mooney S Publication Guidelines for Quality Improvement Studies in Health Care: Evolution of the SQUIRE Project J Gen Intern Med 2008 10.1007/s11606-008-0797-4 PMC2596523 18830766

