# Supplementary material for: Identifying quality improvement intervention publications - A comparison of electronic search strategies
Source: Implement Sci. 2011 Aug 1;6:85. doi: 10.1186/1748-5908-6-85 (PMC3170235; doi:10.1186/1748-5908-6-85)
Supplement: Additional file 1 — Appendix 1. Reference sets. [file 1748-5908-6-85-S1.DOC]

# Additional File 1

# Appendix Reference Sets

## Reference Set #1: AHRQ

1. Beyer, M.; Gerlach, F. M.; Flies, U.; Grol, R.; Krol, Z.; Munck, A.; Olesen, F.; O'Riordan, M.; Seuntjens, L., and Szecsenyi, J. The development of quality circles/peer review groups as a method of quality improvement in Europe. Results of a survey in 26 European countries. Fam Pract. 2003 Aug; 20(4):443-51.
Rec #: 1036

2. Chin, M. H.; Cook, S.; Drum, M. L.; Jin, L.; Guillen, M.; Humikowski, C. A.; Koppert, J.; Harrison, J. F.; Lippold, S., and Schaefer, C. T. Improving diabetes care in midwest community health centers with the health disparities collaborative. Diabetes Care. 2004 Jan; 27(1):2-8.
Rec #: 1032

3. Cretin, S.; Shortell, S. M., and Keeler, E. B. An evaluation of collaborative interventions to improve chronic illness care. Framework and study design. Eval Rev. 2004 Feb; 28(1):28-51.
Rec #: 1023

4. Feifer, C.; Fifield, J.; Ornstein, S.; Karson, A. S.; Bates, D. W.; Jones, K. R., and Vargas, P. A. From research to daily clinical practice: what are the challenges in "translation"? Jt Comm J Qual Saf. 2004 May; 30(5):235-45.
Rec #: 1027

5. Feifer, C. and Ornstein, S. M. Strategies for increasing adherence to clinical guidelines and improving patient outcomes in small primary care practices. Jt Comm J Qual Saf. 2004 Aug; 30(8):432-41.
Rec #: 1078

6. Finkelstein, J. A.; Lozano, P.; Streiff, K. A.; Arduino, K. E.; Sisk, C. A.; Wagner, E. H.; Weiss, K. B., and Inui, T. S. Clinical effectiveness research in managed-care systems: lessons from the Pediatric Asthma Care PORT. Patient Outcomes Research Team. Health Serv Res. 2002 Jun; 37(3):775-89.
Rec #: 1026

7. Frayne, S. M.; Skinner, K. M.; Sullivan, L. M., and Freund, K. M. Sexual assault while in the military: violence as a predictor of cardiac risk? Violence Vict. 2003 Apr; 18(2):219-25.
Rec #: 1025

8. Horbar, J. D.; Carpenter, J. H.; Buzas, J.; Soll, R. F.; Suresh, G.; Bracken, M. B.; Leviton, L. C.; Plsek, P. E., and Sinclair, J. C. Collaborative quality improvement to promote evidence based surfactant for preterm infants: a cluster randomised trial. BMJ. 2004 Oct 30; 329(7473):1004.
Rec #: 1040

9. Horbar JD, Carpenter JH, Buzas J, Soll RF, Suresh G, Bracken MB, Leviton LC, Plsek PE, Sinclair JC; Vermont Oxford Network. Timing of initial surfactant treatment for infants 23 to 29 weeks' gestation: is routine practice evidence based? Pediatrics. 2004 Jun; 113(6):1593-602.
Rec #: 1024

10. Jones, K. R.; Fink, R.; Pepper, G.; Hutt, E.; Vojir, C. P.; Scott, J.; Clark, L., and Mellis, K. Improving nursing home staff knowledge and attitudes about pain. Gerontologist. 2004 Aug; 44(4):469-78.
Rec #: 1028

11. Landon, B. E.; Wilson, I. B.; McInnes, K.; Landrum, M. B.; Hirschhorn, L.; Marsden, P. V.; Gustafson, D., and Cleary, P. D. Effects of a quality improvement collaborative on the outcome of care of patients with HIV infection: the EQHIV study. Ann Intern Med. 2004 Jun 1; 140(11):887-96.
Rec #: 1031

12. Leviton, L. C.; Baker, S.; Hassol, A., and Goldenberg, R. L. An exploration of opinion and practice patterns affecting low use of antenatal corticosteroids. Am J Obstet Gynecol. 1995 Jul; 173(1):312-6.
Rec #: 1021

13. Leviton, L. C.; Goldenberg, R. L.; Baker, C. S.; Schwartz, R. M.; Freda, M. C.; Fish, L. J.; Cliver, S. P.; Rouse, D. J.; Chazotte, C.; Merkatz, I. R., and Raczynski, J. M. Methods to encourage the use of antenatal corticosteroid therapy for fetal maturation: a randomized controlled trial. JAMA. 1999 Jan 6; 281(1):46-52.
Rec #: 1035

14. Lozano, P.; Finkelstein, J. A.; Carey, V. J.; Wagner, E. H.; Inui, T. S.; Fuhlbrigge, A. L.; Soumerai, S. B.; Sullivan, S. D.; Weiss, S. T., and Weiss, K. B. A multisite randomized trial of the effects of physician education and organizational change in chronic-asthma care: health outcomes of the Pediatric Asthma Care Patient Outcomes Research Team II Study. Arch Pediatr Adolesc Med. 2004 Sep; 158(9):875-83.
Rec #: 1029

15. Margolis, P. A.; Lannon, C. M.; Stuart, J. M.; Fried, B. J.; Keyes-Elstein, L., and Moore, D. E. Jr. Practice based education to improve delivery systems for prevention in primary care: randomised trial. BMJ. 2004 Feb 14; 328(7436):388.
Rec #: 1030

16. Margolis, P. A.; Stevens, R.; Bordley, W. C.; Stuart, J.; Harlan, C.; Keyes-Elstein, L., and Wisseh, S. From concept to application: the impact of a community-wide intervention to improve the delivery of preventive services to children. Pediatrics. 2001 Sep; 108(3):E42.
Rec #: 1033

17. Murff, H. J.; Gandhi, T. K.; Karson, A. K.; Mort, E. A.; Poon, E. G.; Wang, S. J.; Fairchild, D. G., and Bates, D. W. Primary care physician attitudes concerning follow-up of abnormal test results and ambulatory decision support systems. Int J Med Inform. 2003 Sep; 71(2-3):137-49.
Rec #: 1018

18. Ornstein, S.; Jenkins, R. G.; Nietert, P. J.; Feifer, C.; Roylance, L. F.; Nemeth, L.; Corley, S.; Dickerson, L.; Bradford, W. D., and Litvin, C. A multimethod quality improvement intervention to improve preventive cardiovascular care: a cluster randomized trial. Ann Intern Med. 2004 Oct 5; 141(7):523-32.
Rec #: 1038

19. Ozer, E. M.; Adams, S. H.; Gardner, L. R.; Mailloux, D. E.; Wibbelsman, C. J., and Irwin, C. E. Jr. Provider self-efficacy and the screening of adolescents for risky health behaviors. J Adolesc Health. 2004 Aug; 35(2):101-7.
Rec #: 1019

20. Phillips, L. S.; Hertzberg, V. S.; Cook, C. B.; El-Kebbi, I. M.; Gallina, D. L.; Ziemer, D. C.; Miller, C. D.; Doyle, J. P.; Barnes, C. S.; Slocum, W.; Lyles, R. H.; Hayes, R. P.; Thompson, D. N.; Ballard, D. J.; McClellan, W. M., and Branch, W. T. Jr. The Improving Primary Care of African Americans with Diabetes (IPCAAD) project: rationale and design. Control Clin Trials. 2002 Oct; 23(5):554-69.
Rec #: 1022

21. Schoenbaum, M.; Unutzer, J.; Sherbourne, C.; Duan, N.; Rubenstein, L. V.; Miranda, J.; Meredith, L. S.; Carney, M. F., and Wells, K. Cost-effectiveness of practice-initiated quality improvement for depression: results of a randomized controlled trial. JAMA. 2001 Sep 19; 286(11):1325-30.
Rec #: 1039

22. Shafer, M. A.; Tebb, K. P.; Pantell, R. H.; Wibbelsman, C. J.; Neuhaus, J. M.; Tipton, A. C.; Kunin, S. B.; Ko, T. H.; Schweppe, D. M., and Bergman, D. A. Effect of a clinical practice improvement intervention on Chlamydial screening among adolescent girls. JAMA. 2002 Dec 11; 288(22):2846-52.
Rec #: 1037

23. Solberg, L. I.; Kottke, T. E.; Brekke, M. L., and Magnan, S. Improving prevention is difficult. Eff Clin Pract. 2000 May-2000 Jun 30; 3(3):153-5.
Rec #: 1041

24. Solberg, L. I.; Kottke, T. E.; Brekke, M. L.; Magnan, S.; Davidson, G.; Calomeni, C. A.; Conn, S. A.; Amundson, G. M., and Nelson, A. F. Failure of a continuous quality improvement intervention to increase the delivery of preventive services. A randomized trial. Eff Clin Pract. 2000 May-2000 Jun 30; 3(3):105-15.
Rec #: 1034

25. Vargas, P. A.; Simpson, P. M.; Gary Wheeler, J.; Goel, R.; Feild, C. R.; Tilford, J. M., and Jones, S. M. Characteristics of children with asthma who are enrolled in a Head Start program. J Allergy Clin Immunol. 2004 Sep; 114(3):499-504.
Rec #: 1020

## Reference Set #2: SQUIRE

1. Agency for Healthcare Research and Quality. Strategies for Improving Minority Healthcare Quality: U.S. Department of Health and Human Services ; 2004 Jan.
Rec #: 180

2. Beyth, R. J.; Quinn, L., and Landefeld, C. S. A multicomponent intervention to prevent major bleeding complications in older patients receiving warfarin. A randomized, controlled trial. Ann Intern Med. 2000 Nov 7; 133(9):687-95.
Rec #: 40

3. Cunliffe, A. L.; Gladman, J. R.; Husbands, S. L.; Miller, P.; Dewey, M. E., and Harwood, R. H. Sooner and healthier: a randomised controlled trial and interview study of an early discharge rehabilitation service for older people. Age Ageing. 2004 May; 33(3):246-52.
Rec #: 190

4. Epstein, R. M.; Alper, B. S., and Quill, T. E. Communicating evidence for participatory decision making. JAMA. 2004 May 19; 291(19):2359-66.
Rec #: 170
Notes: GENERAL NOTE: KIE: KIE Bib: Professional Patient Relationship

5. Fahey, T.; Schroeder, K., and Ebrahim, S. Educational and organisational interventions used to improve the management of hypertension in primary care: a systematic review. Br J Gen Pract. 2005 Nov; 55(520):875-82.
Rec #: 260

6. Fellowes, D.; Wilkinson, S., and Moore, P. Communication skills training for health care professionals working with cancer patients, their families and/or carers. Cochrane Database Syst Rev. 2007; (3).
Rec #: 290

7. Gibson, P. G. and Powell, H. Written action plans for asthma: an evidence-based review of the key components. Thorax. 2004 Feb; 59(2):94-9.
Rec #: 270

8. Hillman, K.; Chen, J.; Cretikos, M.; Bellomo, R.; Brown, D.; Doig, G.; Finfer, S., and Flabouris, A. Introduction of the medical emergency team (MET) system: a cluster-randomised controlled trial. Lancet. 2005 Jun 18-2005 Jun 24; 365(9477):2091-7.
Rec #: 30
Notes: CORPORATE NAME: MERIT study investigators

9. Landefeld, C. S. and Anderson, P. A. Guideline-based consultation to prevent anticoagulant-related bleeding. A randomized, controlled trial in a teaching hospital. Ann Intern Med. 1992 May 15; 116(10):829-37.
Rec #: 50

10. Landefeld, C. S.; Palmer, R. M.; Kresevic, D. M.; Fortinsky, R. H., and Kowal, J. A randomized trial of care in a hospital medical unit especially designed to improve the functional outcomes of acutely ill older patients. N Engl J Med. 1995 May 18; 332(20):1338-44.
Rec #: 60

11. Landon, B. E.; Wilson, I. B.; McInnes, K.; Landrum, M. B.; Hirschhorn, L.; Marsden, P. V.; Gustafson, D., and Cleary, P. D. Effects of a quality improvement collaborative on the outcome of care of patients with HIV infection: the EQHIV study. Ann Intern Med. 2004 Jun 1; 140(11):887-96.
Rec #: 70

12. Loeb, M.; Carusone, S. C.; Goeree, R.; Walter, S., and et al. Effect of a Clinical Pathway to Reduce Hospitalizations in Nursing Home Residents With Pneumonia: A Randomized Controlled Trial. 2006; 295(21):2503-10.
Rec #: 250

13. McClellan, W. M.; Millman, L.; Presley, R.; Couzins, J., and Flanders, W. D. Improved diabetes care by primary care physicians: results of a group-randomized evaluation of the Medicare Health Care Quality Improvement Program (HCQIP). J Clin Epidemiol. 2003 Dec; 56(12):1210-7.
Rec #: 200

14. Mills, P. D.; Neily, J.; Mims, E.; Burkhardt, M. E., and Bagian, J. Improving the bar-coded medication administration system at the Department of Veterans Affairs. Am J Health Syst Pharm. 2006 Aug 1; 63(15):1442-7.
Rec #: 150

15. Mittman, B. S. Creating the evidence base for quality improvement collaboratives. Ann Intern Med. 2004 Jun 1; 140(11):897-901.
Rec #: 130

16. Mooney, S. E.; Ogrinc, G., and Steadman, W. Improving emergency caesarean delivery response times at a rural community hospital. Qual Saf Health Care. 2007 Feb; 16(1):60-6.
Rec #: 230

17. Neily, J.; Howard, K.; Quigley, P., and Mills, P. D. One-year follow-up after a collaborative breakthrough series on reducing falls and fall-related injuries. Jt Comm J Qual Patient Saf. 2005 May; 31(5):275-85.
Rec #: 140

18. Pronovost, P.; Needham, D.; Berenholtz, S.; Sinopoli, D.; Chu, H.; Cosgrove, S.; Sexton, B.; Hyzy, R.; Welsh, R.; Roth, G.; Bander, J.; Kepros, J., and Goeschel, C. An intervention to decrease catheter-related bloodstream infections in the ICU. N Engl J Med. 2006 Dec 28; 355(26):2725-32.
Rec #: 120

19. Rollow, W.; Lied, T. R.; McGann, P.; Poyer, J.; LaVoie, L.; Kambic, R. T.; Bratzler, D. W.; Ma, A.; Huff, E. D., and Ramunno, L. D. Assessment of the Medicare quality improvement organization program. Ann Intern Med. 2006 Sep 5; 145(5):342-53.
Rec #: 110

20. Samore, M. H.; Bateman, K.; Alder, S. C.; Hannah, E.; Donnelly, S.; Stoddard, G. J.; Haddadin, B.; Rubin, M. A.; Williamson, J.; Stults, B.; Rupper, R., and Stevenson, K. Clinical decision support and appropriateness of antimicrobial prescribing: a randomized trial. JAMA. 2005 Nov 9; 294(18):2305-14.
Rec #: 220

21. Shea, S.; Weinstock, R. S.; Starren, J.; Teresi, J., and et.al. A Randomised Trial Comparing Telemedicine Case Management with Usual Care in Older, Ethically Diverse, Medically Underserved Patients with Diabetes Mellitus. J Am Med Inform Assoc. 2006; 13:40-51.
Rec #: 100

22. Shojania, K. G.; Ranji, S. R.; McDonald, K. M.; Grimshaw, J. M.; Sundaram, V.; Rushakoff, R. J., and Owens, D. K. Effects of quality improvement strategies for type 2 diabetes on glycemic control: a meta-regression analysis. JAMA. 2006 Jul 26; 296(4):427-40.
Rec #: 80

23. Simpson, S. H.; Eurich, D. T.; Majumdar, S. R.; Padwal, R. S.; Tsuyuki, R. T.; Varney, J., and Johnson, J. A. A meta-analysis of the association between adherence to drug therapy and mortality. BMJ. 2006 Jul 1; 333(7557):15.
Rec #: 280

24. Unutzer, J.; Katon, W.; Callahan, C. M.; Williams, J. W. Jr; Hunkeler, E.; Harpole, L.; Hoffing, M.; Della Penna, R. D.; Noel, P. H.; Lin, E. H.; Arean, P. A.; Hegel, M. T.; Tang, L.; Belin, T. R.; Oishi, S., and Langston, C. Collaborative care management of late-life depression in the primary care setting: a randomized controlled trial. JAMA. 2002 Dec 11; 288(22):2836-45.
Rec #: 10
Notes: CORPORATE NAME: IMPACT Investigators. Improving Mood-Promoting Access to Collaborative Treatment

25. Weeks, W. B.; Mills, P. D.; Waldron, J.; Brown, S. H.; Speroff, T., and Coulson, L. R. A model for improving the quality and timeliness of compensation and pension examinations in VA facilities. J Healthc Manag. 2003 Jul-2003 Aug 31; 48(4):252-61; discussion 262.
Rec #: 160

26. Wells, K. B.; Sherbourne, C.; Schoenbaum, M.; Duan, N.; Meredith, L.; Unutzer, J.; Miranda, J.; Carney, M. F., and Rubenstein, L. V. Impact of disseminating quality improvement programs for depression in managed primary care: a randomized controlled trial. JAMA. 2000 Jan 12; 283(2):212-20.
Rec #: 240

27. Werner, R. M. and Bradlow, E. T. Relationship between Medicare's hospital compare performance measures and mortality rates. JAMA. 2006 Dec 13; 296(22):2694-702.
Rec #: 20

28. Wu, J. Y.; Leung, W. Y.; Chang, S.; Lee, B.; Zee, B.; Tong, P. C., and Chan, J. C. Effectiveness of telephone counselling by a pharmacist in reducing mortality in patients receiving polypharmacy: randomised controlled trial. BMJ. 2006 Sep 9; 333(7567):522.
Rec #: 90

29. Yealy, D. M.; Auble, T. E.; Stone, R. A.; Lave, J. R.; Meehan, T. P.; Graff, L. G.; Fine, J. M.; Obrosky, D. S.; Mor, M. K.; Whittle, J., and Fine, M. J. Effect of increasing the intensity of implementing pneumonia guidelines: a randomized, controlled trial. Ann Intern Med. 2005 Dec 20; 143(12):881-94.
Rec #: 210

## Reference Set #3: EPOC

1. Jansa, M., M. Vidal, J. Viaplana, I. Levy, I. Conget, R. Gomis and E. Esmatjes (2006). "- Telecare in a structured therapeutic education programme addressed to patients with type 1 diabetes and poor metabolic control." - 2006 Oct; 74(- 1): - 26

2. Katon, W., J. Russo, C. Sherbourne, M. B. Stein, M. Craske, M. Y. Fan and P. Roy-Byrne (2006). "- Incremental cost-effectiveness of a collaborative care intervention for panic disorder." **- 36**(- 3): - 353-363

3. Florin, J., A. Ehrenberg and M. Ehnfors (2005). "- Quality of nursing diagnoses: evaluation of an educational intervention." **- 16**(- 2): - 33-43

4. Wells, K., C. Sherbourne, N. Duan, J. Unutzer, J. Miranda, M. Schoenbaum, S. L. Ettner, L. S. Meredith and L. Rubenstein (2005). "- Quality improvement for depression in primary care: do patients with subthreshold depression benefit in the long run?" **- 162**(- 6): - 1149-1157

5. Taylor, K. I., K. M. Oberle, R. A. Crutcher and P. G. Norton (2005). "- Promoting health in type 2 diabetes: nurse-physician collaboration in primary care." **- 6**(- 3): - 207-215

6. Akashi, H., T. Yamada, E. Huot, K. Kanal and T. Sugimoto (2004). "- User fees at a public hospital in Cambodia: effects on hospital performance and provider attitudes." **- 58**(- 3): - 553-564

7. Gilliam, F. G., A. J. Fessler, G. Baker, V. Vahle, J. Carter and H. Attarian (2004). "- Systematic screening allows reduction of adverse antiepileptic drug effects: a randomized trial. see comment." **- 62**(- 1): - 23-27

8. Scott, I. A., C. P. Denaro, A. C. Hickey, C. Bennett, A. M. Mudge, D. C. Sanders, J. Thiele and J. L. Flores (2004). "- Optimising care of acute coronary syndromes in three Australian hospitals." **- 16**(- 4): - 275-284

9. Vollenbroek-Hutten, M. M., H. J. Hermens, D. Wever, M. Gorter, J. Rinket and M. J. Ijzerman (2004). "- Differences in outcome of a multidisciplinary treatment between subgroups of chronic low back pain patients defined using two multiaxial assessment instruments: the multidimensional pain inventory and lumbar dynamometry." **- 18**(- 5): - 566-579

10. Reiber, G. E., D. Au, M. McDonell and S. D. Fihn (2004). "- Diabetes quality improvement in Department of Veterans Affairs Ambulatory Care Clinics: a group-randomized clinical trial." **- 27**(- Supp2): - B61-B68

11. Goff, D. C., Jr., L. Gu, L. K. Cantley, D. J. Sheedy and S. J. Cohen (2003). "- Quality of care for secondary prevention for patients with coronary heart disease: results of the Hastening the Effective Application of Research through Technology (HEART) trial. see comment." **- 146**(- 6): - 1045-1051

12. McClellan, W. M., L. Millman, R. Presley, J. Couzins and W. D. Flanders (2003). "- Improved diabetes care by primary care physicians: results of a group-randomized evaluation of the Medicare Health Care Quality Improvement Program (HCQIP)." **- 56**(- 12): - 1210-1217

13. Ferguson, T. B. (2003). "- Continuous quality improvement in medicine: validation of a potential role for medical specialty societies." **- 1**(- 4): - 264-272

14. Scott, J., A. Thorne and P. Horn (2002). "- Quality improvement report: Effect of a multifaceted approach to detecting and managing depression in primary care." **- 325**(- 7370): - 951-954

15. Smyrnios, N. A., A. Connolly, M. M. Wilson, F. J. Curley, C. T. French, S. O. Heard and R. S. Irwin (2002). "- Effects of a multifaceted, multidisciplinary, hospital-wide quality improvement program on weaning from mechanical ventilation." **- 30**(- 6): - 1224-1230

16. Geboers, H., H. Mokkink, M. P. van, H. en Hoogen, W. en Bosch and R. Grol (2001). "- Continuous quality improvement in small general medical practices: the attitudes of general practitioners and other practice staff." **- 13**(- 5): - 391-397

17. Lichtman, J. H., S. A. Roumanis, M. J. Radford, M. S. Riedinger, S. Weingarten and H. M. Krumholz (2001). "- Can practice guidelines be transported effectively to different settings? Results from a multicenter interventional study." **- 27**(- 1): - 42-53

18. McBride, P., G. Underbakke, M. B. Plane, K. Massoth, R. Brown, L. I. Solberg, L. Ellis, H. G. Schrott, K. Smith, T. Swanson, E. Spencer, G. Pfeifer and A. Knox (2000). "- Improving prevention systems in primary care practices: the Health Education and Research Trial (HEART) see comments." **- 49**(- 2): - 115-125

19. Moskowitz, H., J. Sunshine, D. Grossman, L. Adams and L. Gelinas (2000). "- The effect of imaging guidelines on the number and quality of outpatient radiographic examinations." **- 175**(- 1): - 9-15

20. Kim, C. S., R. J. Kristopaitis, E. Stone, M. Pelter, M. Sandhu and S. R. Weingarten (1999). "- Physician education and report cards: do they make the grade? results from a randomized controlled trial." **- 107**(- 6): - 556-560

21. Emond, S. D., P. G. Woodruff, E. Y. Lee, A. K. Singh and C. A. Camargo, Jr. (1999). "- Effect of an emergency department asthma program on acute asthma care see comments." **- 34**(- 3): - 321-325

22. Ram, P., C. van der Vleuten, J. J. Rethans, R. Grol and K. Aretz (1999). "- Assessment of practicing family physicians: comparison of observation in a multiple-station examination using standardized patients with observation of consultations in daily practice." **- 74**(- 1): - 62-69

23. Curley, C., J. E. McEachern and T. Speroff (1998). "- A firm trial of interdisciplinary rounds on the inpatient medical wards: an intervention designed using continuous quality improvement." **- 36**(- 8 Suppl): - AS4-12

24. O'Connor, P. J., W. A. Rush, J. Peterson, P. Morben, L. Cherney, C. Keogh and S. Lasch (1996). "- Continuous quality improvement can improve glycemic control for HMO patients with diabetes." **- 5**(- 9): - 502-506

25. Saturno, P. J. (1995). "- Training health professionals to implement quality improvement activities. Results of a randomized controlled trial after one year of follow-up." **- 7**(- 2): - 119-126

26. Anderson, R. B., R. D. Needleman, R. A. Gatter, R. P. Andrews and J. A. Scarola (1988). "- Patient outcome following inpatient vs outpatient treatment of rheumatoid arthritis." **- 15**(- 4): - 556-560
