# Supplementary material for: Identifying quality improvement intervention publications - A comparison of electronic search strategies
Source: Implement Sci. 2011 Aug 1;6:85. doi: 10.1186/1748-5908-6-85 (PMC3170235; doi:10.1186/1748-5908-6-85)
Supplement: Additional file 2 — Appendix table. Application of published validated search strategies. [file 1748-5908-6-85-S2.DOC]

# Additional File 2

# Appendix application of published validated search strategies

**Appendix table 1: A**pplication of published validated search strategies

| Strategy | Description | Search terms and databases searched | Total retrieval rate (Yield) | Recall AHRQ set  (n=25) | Recall SQUIRE set  (n=29) | Recall EPOC set  (n=26) | Across sets | Recall:Yield Ratio |
| --- | --- | --- | --- | --- | --- | --- | --- | --- |
| Balas et al. [7]  #1 | Intervention text words | (home care OR patient education OR patient reminder OR physician education OR physician reminder OR provider feedback OR telephone follow-up).mp.  (MEDLINE, Ovid) | 88,079 | n/a | n/a | n/a | n/a | n/a |
| Balas et al. [7]  #2 | Intervention MeSH terms | (home care services OR patient education OR reminder systems OR education, medical, continuing OR reminder system OR feedback OR telephone).sh.  (MEDLINE, Ovid) | 67,563 | n/a | n/a | n/a | n/a | n/a |
| Balas et al. [7]  #3 | Intervention text words + effect variables | 1 (home care OR patient education OR patient reminder OR physician education OR physician reminder OR provider feedback OR telephone follow-up).mp.  2 (c-section rate OR cost of care OR follow-up visits OR hospitalization rate OR immunization rate OR length of stay OR number of prescriptions).mp.  3 1 AND 2  (MEDLINE, Ovid) | 1,660 | 0 | 0 | 0 | 0% | 0 |
| Balas et al. [7]  #4 | Intervention MeSH terms + effect variables | 1 (home care services OR patient education OR reminder systems OR education, medical, continuing OR reminder system OR feedback OR telephone).sh.  2 (Cesarean section sn OR health care costs OR follow-up studies OR hospitalization sn OR immunization sn OR immunization OR length of stay OR prescriptions, drug sn OR prescriptions, drug).sh.  3 1 AND 2  (MEDLINE, Ovid) | 2,132 | 3 | 0 | 0 | 4% | 0.00188 |
| QI Hedges [11]  #1 | Best sensitivity, all QI | exp health services administration/ OR random:.mp. OR review.pt. OR compare:.tw.  (MEDLINE, Ovid) | 4,175,222 | n/a | n/a | n/a | n/a | n/a |
| QI Hedges [11]  #2 | Best sensitivity, QI pass for methods | effectivess.tw. OR journal.mp. OR MEDLINE.tw. OR random:.tw.  (MEDLINE, Ovid) | 15,681,694 | n/a | n/a | n/a | n/a | n/a |
| QI Hedges [11]  #3 | Optimization of sensitivity and specificity, all Q | random:.ti,ab. OR educat:.tw. OR exp patient care management/  (MEDLINE, Ovid) | 933,460 | n/a | n/a | n/a | n/a | n/a |
| QI Hedges [11]  #4 | Optimization of sensitivity and specificity, QI pass for method | (control: trial: OR journal).mp. OR MEDLINE.tw. OR random: trial:.tw.  (MEDLINE, Ovid) | 15,691,611 | n/a | n/a | n/a | n/a | n/a |

Notes: Search period: database inception to January 2008; mp: term present in the title, original title, abstract, name of substance word, subject heading word, unique identifier; .sh indicates MeSH subject heading (not exploded); AND, OR: Boolean operators; n/a: not available, only strategies with yields <50,000 were followed up; Balas et al. did not suggest specific search strategies but tested individual text words and MeSH terms which we combined to test the filter performance
